# Supplementary material for: Content-rich biological network constructed by mining PubMed abstracts
Source: BMC Bioinformatics. 2004 Oct 8;5:147. doi: 10.1186/1471-2105-5-147 (PMC528731; doi:10.1186/1471-2105-5-147)
Supplement: Additional File 2 — The original results of the above study (non-essential files are deleted to keep the file size under the limit set by BMC bioinformatics). [file 1471-2105-5-147-S2.bz2 › chilibotAdditionalFile2/dip05/99ID7768895E426/html/STXBP2_SYN3.html]

 


 **STXBP2** and **SYN3** 
  
Found 1 abstracts in PubMed, retrieved 1.  
 

 What does Google say? 
 PDF only 
| .edu only 

---

**Interactive relationship** (e.g. stimulation, inhibition, etc)

**Neutral relationship**- This implies that Munc18 2  [ **STXBP2** ]  function in apical membrane trafficking involves aspects independent of the syntaxin 3  [ **SYN3** ]  interaction.  Ref: 10788461 J Biol Chem, 2000
- The in vitro and in vivo binding patterns were highly similar, and the association of the Munc18 2  [ **STXBP2** ]  variants with syntaxin 3  [ **SYN3** ]  correlated well with their ability to displace SNAP 23 from syntaxin 3  [ **SYN3** ]  complexes when overexpressed in Caco 2 cells.  Ref: 10788461 J Biol Chem, 2000
- Munc18 2  [ **STXBP2** ]  is a Sec1 homologue enriched in epithelial cells and forms a complex with syntaxin 3  [ **SYN3** ] , a t SNARE localized to the apical plasma membrane.  Ref: 10788461 J Biol Chem, 2000

**Non-interactive relationship** (e.g. studied together, co-existance, homology, etc.)

- Even the Munc18 2  [ **STXBP2** ]  mutants that do not detectably bind syntaxin 3  [ **SYN3** ]  were membrane associated in Caco 2 cells, suggesting that the syntaxin interaction is not the sole determinant of Sec1 protein membrane attachment.  Ref: 10788461 J Biol Chem, 2000
- Munc18 2  [ **STXBP2** ] , a functional partner of syntaxin 3  [ **SYN3** ] , controls apical membrane trafficking in epithelial cells.  Ref: 10788461 J Biol Chem, 2000
